# The Hospital

**Published:** 1918-01-19

**Authors:** 


THE
### INSTITUTIONAL WORKER
**Being a Special Supplement to "The Hospital"**

### OUR BUREAU OF INFORMATION.

### Rules for Correspondents.

1. Every letter must be accompanied by the coupon to be cut from the back cover (inside page) of THE HOSPITAL, current issue, and must contain the name and address of the correspondent with pseudonym for publication if desired. Replies by post cannot be given save under exceptional circumstances at the Editor's discretion.

2. Letters from Approved Homes in reply to special needs published in the Bureau should state terms and full particulars, and be sent prepaid under cover to the Editor of the Bureau with name written across coupon for identification.

3. Proprietors of Homes which have not yet been entered on the List of Approved Homes, but have spare accommodation likely to suit special needs, are invited to write for an application form for registration The fee for registration, which includes two announcements of the Home in the Bureau and other privileges, is 10s.

4. All communications to be addressed to the Editor of THE HOSPITAL, 28 Southampton Street, Strand, London, W.C. 2, and marked "Bureau of Information."

### INSTITUTION FACTS AND FIGURES.

### The Ontario Military Hospital, Orpington.

[We have pleasure in publishing the following spontaneous testimony from an Australian patient in the Ontario Military Hospital, Orpington. It cannot fail to be a satisfaction both to those who have supplied the hospital and to the authorities who are responsible for the efficiency of its upkeep to realise, that, in the hospital treatment care and comfort, and the opportunities for knowledge of each other thus afforded to members of the Dominions, they are helping immeasurably to strengthen the links which bind together the various units of our great Empire.]

BEFORE the war an Australian journalist, now a soldier in the army of that nation, it has been my good fortune, when wounded in the third battle of Ypres (the Menin Road affair), to be sent to the Ontario Military Hospital at Orpington, Kent.

It is only fair to other military hospitals to say that this one has the inestimable advantage over many of them in being built to order. It is a very different proposition, having to make the best of whatever conveniences there happen to be, and having all the conveniences that the science of hospital management and equipment provide. In this case, utilitarianism has been raised to a fine art.

The lighting and heating are furnished by three high-speed compound engines, and some idea of the extent of the installation will be gathered from the statement that it comprises nineteen miles of steam piping and heating apparatus. Over one hundred tons of coal are used in one week in providing forty thousand gallons of steam daily, which is necessary for the heating of all the buildings, the water for bathing, for the numerous cookers. and the disinfecting chamber.

The light-green walls and white ceilings of the hospital wards give a pleasing if subdued tone, and the inverted-bowl shades of the numerous electric lamps dispense a light from which all glare has been removed. The most striking feature of the wards is that whereas one wall has an ordinary amount of window space, the other is windowed down halfway to the floor for the whole length of the ward—*i.e.*, there is as much window as wall. This, with the sensible latitude allowed by the genial nursing sisters for larking amongst the "boys," actually gives the place the air of a school dormitory rather than that of a military hospital.

As with all battlers in all places, the food problem has first place in a soldier's regard. Kingdoms may rise or fall, dynasties topple over in a night, his politicians may rave themselves into delirium, reputations may be made or lost, the enemy may strafe, and his own officers may say what they please, so long as they don't call him late for a decent dinner. His food is a more prolific cause of discontent than anything else, and I, as an old soldier, had come to despair of seeing food cooked properly in bulk, or good tea being brewed by the gallon. Yet here it is done.

Five hundred "up-patients" sit down in the dining-hall to porridge and milk, with hot mincemeat, fried bacon, or liver, for breakfast; roast meat four days a week, stews twice a week, and fish once a week for dinner, with milk puddings, fruit, or custard of some kind, and some hot meat or minced fish for tea, with unlimited bread-and-butter at every meal; sometimes jam and cheese as an alternative at tea-time. Supper in the shape of bread-and-butter and cocoa is on at seven o'clock. The tea that is brewed is made in a steam-heated tank, which, together with the fact that the hospital draws some of the best water in England, accounts for the excellence of the beverage so prized by tea-toping Australians.

Bed patients are served with their food by a system as perfect as is humanly possible, and these get extras such as chicken, other vegetables than potatoes, and various puddings.

Excellent bread is baked in the large, modern Collins ovens, 2,500 lb. daily being the average batch. It is impossible for me to go into much detail, but, from the moment the food leaves the hands of the chief cook to the time when one sits before it on a solid, stable, comfortable form, it is handled swiftly and efficiently by a highly-trained staff, and it would be difficult to find a reasonable complaint from any of the 2,200 patients.

If a patient requires anything over the daily menu, he is at liberty to purchase goods from an exceedingly well-stocked canteen, whose prices, governed by the Army and Navy Canteen Board, compare more than favourably with outside ones. An unselfish, untiring manageress, seconded by a loyal staff, dispenses refreshments and other goods to the patients and their friends who come to visit them.

In the recreation-hall adjoining the canteen, cinema shows and concerts are held throughout the week, sufficient generous spirits being found to provide a "show" of some kind on six nights a week.

Twenty-five per cent. leave is given to visit the surrounding pretty country, and though there would seem to be no argument against general leave being granted, I am told that it once proved impracticable, and that as much freedom is allowed as is consistent with the smooth working of the institution. Day leave to London is granted to an average of three per ward, and is much appreciated. The frank courtesy of all grades of the staff is a reflex of the generous outlook of a sister nation, and an indication that the links that bind us are not of expediency's forging, but were made in the fire of mutual idealism. The cost of upkeep for this institution must be considerable, and I fancy that a few at least of the taxpayers in Ontario have wondered whether anyone was getting the value of the cash nexus. I think that the soldiers are, and more, in that here and in similar temples they also muster those moral resources that make the possessors practical idealists, and that will show them in the end as more than conquerors in the world's fight.

### JANUARY QUESTIONS.

### 1. Extending a Hospital Engineer's Work.

In what ways can a hospital engineer be useful in addition to his routine work of attending to the heating apparatus?

### 2. A Domestic Matter in a Nurses' Home.

Describe the cleanest, quickest, and most careful method of washing and drying crockery in a nurses' home to accommodate 180 persons.

### RULES.

The following rules must be observed:—

1. Contributions must be written on one side of the paper. Brevity and terseness are desirable features. The MSS. must bear the name and address of the sender and be accompanied by coupon to be cut from the back cover (inside page) of the current issue of The Hospital. A pseudonym must be chosen if the name is not to be published.
2. Contributions must be addressed to the Editor of The Hospital, Institutional Worker Supplement, 28 & 29 Southampton Street, Strand, London, W.C. 2, should reach him before the end of the current month, and be marked in the left-hand corner "Facts and Figures."

**A minimum payment of Five Shillings will be made for each published answer to the January Questions.**

### QUESTIONS INVITED.

In connection with our Question Box, we offer every month five shillings each for the two best questions which are sent in for consideration. The questions may be on any institutional subject and concern any department, from the secretary's to the porter's, or the matron's to the domestic staff; the one essential is that they must be practical and deal with points that have a definite relation to hospital or institutional administration, upkeep, finance, or management. Points relating to artificers' work, housekeeping, the laundry, out-patients, and other practical matters will be welcome. It is hoped that thus, when difficult or doubtful points arise in the course of their work, institutional workers will be encouraged to put them in the form of a question and send them to the Editor, The Hospital Bureau of Information, 28 Southampton Street, Strand, W.C. 2, marked "Question Box."

The best questions will be published in due course and our readers will be given the opportunity of answering them. Thus all institutional workers may be able to co-operate with the view of helping the work and smoothing the difficulties of each other. In short, we want the Question Box of the *Institutional Worker* to be freely used by every worker habitually

### ENQUIRIES AND ANSWERS.

### *THE SICK AND IN NEED.*

#### Home for Chronic Invalid.

We suggest that you write to the Hon. Secretary of the Alexandra Home for Chronic Invalids, St. Peter's Road, St. Leonards-on-Sea, and inquire if there is a vacancy; also give full particulars of the case in question. The Home is for women only from twenty-one years of age. A fairly small weekly payment (in advance) is required.—Molly.

#### Twilight Sleep.

The treatment known as "Twilight Sleep" is undertaken at many nursing homes, providing it is recommended by the patient's doctor. We suggest that you consult your medical adviser, who would be in a position to recommend a suitable home. A coupon should be enclosed with each inquiry. We cannot give postal replies.—E. A.

### *EMPLOYMENT AND TRAINING.*

#### Mothers' Union Offices.

The offices of the Mothers' Union have for some time been in Dean's Yard, Westminster. The two aims of the Union are, firstly, to train their workers on modern lines, recognising that the moral, physical, and spiritual life of the child should each receive due attention; and, secondly, to educate in hygiene the intelligent mother of a higher social station than the one usually reached by a maternity and infant-welfare centre. Write to the Secretary, "Mary Sumner" House Training Scheme, Church House, Westminster, S.W. 1.—St. John.

#### Address of Nurses' Club.

The address of the Imperial Nurses' Club is 137 Ebury Street, London, S.W., which is within ten minutes' walk of Victoria Station. The original rules of the club were that it should be open on Sundays and week-days from 9 a.m. to 10 p.m., and only fully-trained nurses or those in course of training should be eligible. The entrance fee for the former is 5s., and the subscription is 10s. 6d. for one year, 7s. 6d. for six months, and 5s. for one month. For nurses in course of full general training the entrance fee is 2s. 6d., the subscription 7s. 6d. for one year and 5s. for six months. Write to the Lady Superintendent.—Trained Nurse.

#### General Training in Derby.

If you are between the ages of twenty-one and thirty you would possibly be accepted for training at the Derbyshire Royal Infirmary, Derby, providing your other necessary qualifications were satisfactory. The training is for four years, the fourth year being spent in private nursing and in the wards. No premium is required; a small salary is given from the beginning of training. Apply to the Matron.—Sara.

#### Midwifery Training.

Trained nurses are accepted for a four months' course of training in midwifery at Queen Charlotte's Lying-in Hospital, Marylebone Road, N.W. The fee is £25. At the City of London Lying-in Hospital, City Road, E.C., the fee is reduced to £21 for trained nurses, and at the General Lying-in Hospital, York Road, Lambeth, S.E., the fee is twenty-eight guineas. At the last-named institution a personal application to the matron must be made by the intending pupil on any week-day between the hours of 11 a.m. and 1 p.m. A coupon should be enclosed with each inquiry. We cannot give postal replies.—M. T.

### *MISCELLANEOUS.*

#### Apparatus for Electro-Medical Treatment.

Where a direct current is available the cheapest and most reliable and safe apparatus to instal for galvanisation and ionisation is the Galvanoset, made by the Medical Supply Association, of 167/85 Gray's Inn Road, London, W.C. The apparatus can be connected with the main supply by means of a standard electric-light adaptor. The current is conducted through two lamp resistances into a vessel of water by means of two electrodes placed on opposite sides of the vessel. At right angles to these two electrodes is placed a second pair of electrodes; these are for use in connection with the patient. So long as these two electrodes remain at right angles to the supply electrodes no current will pass in the patient's circuit, for in this equidistant position the electrical potential is at zero. If the disc to which the supply terminals are connected is rotated from its right-angle position, any desired current from zero to maximum can be obtained in the patient's circuit by very gradual increases. By rotating the disc in the opposite direction the current can be reversed. Undulating reversing currents can be obtained by rotating the disc from one direction to another. A graduated scale is attached to record the degree of movement of the disc, and a dead beat milliampere-meter is also provided. The Galvanoset is perfectly safe, for it is impossible for the patient to get into direct contact with either the positive or negative side of the main supply. The price is £8 8s.—Electrical Sister.

**The Editor will be glad to receive correspondence and to consider contributions upon all subjects relating to institutional work which affect the welfare of institutional workers.**

